# Multiple pathogens and prostate cancer

**DOI:** 10.1186/s13027-022-00427-1

**Published:** 2022-05-30

**Authors:** James S. Lawson, Wendy K. Glenn

**Affiliations:** School of Biotechnology and Biomolecular Sciences, University of New South Wales, Sydney, 2052 Australia

**Keywords:** Prostate cancer, Infections, Causation, Human papilloma virus, Pathogens

## Abstract

**Background:**

The aim of this review is to consider whether multiple pathogens have roles in prostate cancer.

**Methods:**

We have reviewed case control studies in which infectious pathogens in prostate cancer were compared to normal and benign prostate tissues. We also reviewed additional evidence from relevant published articles.

**Results:**

We confirmed that high risk human papilloma viruses are a probable cause of prostate cancer. We judged *Escherichia coli*, *Cutibacterium acnes*, *Neisseria gonorrhoea*, *Herpes simplex*, Epstein Barr virus and Mycoplasmas as each having possible but unproven roles in chronic prostatic inflammation and prostate cancer. We judged Cytomegalovirus, Chlamydia trachomatis, Trichomonas vaginalis and the Polyoma viruses as possible but unlikely to have a role in prostate cancer.

**Conclusions and actions:**

The most influential cause of prostate cancer appears to be infection induced chronic inflammation. Given the high prevalence of prostate cancer it is important for action to can be taken without waiting for additional conclusive evidence. These include:

1. Encouragement of all boys (as well as girls) to have HPV vaccines
2. The vigorous use of antibiotics to treat all bacterial pathogens identified in the urogenital tract
3. The use of antiviral medications to control herpes infections
4. Education about safe sexual practices

## Introduction

The aim of this review is to consider whether multiple pathogens have roles in prostate cancer. Multiple pathogens have long been hypothesised as an underlying cause of prostate cancer. However, apart from high-risk for cancer human papilloma viruses (HPVs), no specific pathogens have confirmed causal roles.

We have previously shown that high risk for cancer human papilloma viruses have a probable, but not conclusive, causal role in prostate cancer [1]. This is important because of the availability of safe and effective vaccines against HPV infections. In this review we have updated the evidence which may implicate other infectious pathogens.

We consider it is unlikely that any acute infectious pathogens cause prostate cancer. On the other hand, infectious pathogens that cause long term chronic inflammation are likely to have roles in prostate cancer.

### Epidemiology

Prostate cancer develops in 1 in 8 Western men [2]. About 60% of cases occurs in men aged 65 years or older. It is rare in men under the age of 40 years. About 30% of men have undiagnosed prostate cancer at the time of their death, hence the saying “many men die with, rather than from, prostate cancer”. Prostate cancer occurs more frequently in Western than Asian men [2]. When Asian men migrate from low to high risk countries the risk of developing prostate cancer increases [3]. The reason is not known. However, the number of immigrants developing prostate cancer is still lower than that of men in Western countries [4]. This phenomena is also present in breast cancer for Asian women who migrate from low to high risk countries, the risk of breast cancer rapidly increases within two generations to almost equal that of the host country [5].

## Methods

We have conducted a review of selected English language publications listed in PubMed from 1960 to 2021 relevant to infectious pathogens and prostate cancer. Only studies which included controls were reviewed. Any form of selection introduces bias. For this reason the two authors independently selected the studies that were considered. Any differences in the selection were discussed and joint decisions were made. Additional problems in the assessment of the role of specific pathogens in prostate cancer include (1) the variations in outcomes of studies using similar methods in the same populations, (2) contamination of the prostate specimens and (4) the absence of benign or normal prostate controls.

The selection of pathogens for this review was based on the many previous studies of infections and prostate cancer. These pathogens included Human papilloma viruses, Cetabacterium acnes, Herpes viruses including Epstein Barr virus, Neisseria gonorrhoea, Herpes simplex, Epstein Barr virus, Cytomegalovirus, Chlamydia bacteria, Trichomonas bacteria, Mycoplasmas and Polyoma viruses. Case control studies were available for each of these pathogens. Other pathogens, for which no case controls have been conducted, may also have roles in prostate cancer, for example Escherichia coli, fungal prostatitis, mouse mammary tumour virus and human immunodeficiency virus [6, 7].

The use of case control studies for the study of infections and prostate cancer can be misleading. This is because in most studies the non-cancer controls were benign prostate tissues. Chronic infections are common in the prostate and this can negate the comparisons between cancer and controls.

The Bradford Hill criteria have been frequently used for assessing causal roles of pathogens and other agents [8]. These criteria have been immensely influential. They have largely replaced the famous Koch postulates. Over the last 50 years, it has been estimated that over 100,000 published articles have used the Hill criteria [9]. Hill developed nine criteria in the context of his research into the links between tobacco smoking and lung cancer [10]. At that time the role of viruses in various human cancers was not known. In addition, since 1965 there have been major developments in knowledge and technology. It has also been realised that the relevance of the individual criteria vary according to the nature of the pathogen or harmful agent. Accordingly, there has been a need to add and modify the classic Hill criteria. The list of the Hill and extended criteria in some order of importance include:

(1) Identification and history of the candidate pathogen. (2) Epidemiology. (3) Strength of the association between the pathogen and prostate cancer. (4). Temporality (timing) of the association which includes evidence of infection by a pathogen in normal tissues before the development of the cancer. (5). Does exposure to the pathogen lead to infection, oncogenesis and cancer? (6) Experimental evidence, for example, capacity of the pathogen to cause cancer in experimental animals, capacity to infect human cells, ability to transform normal human cells into malignant cells, evidence that a vaccine or therapy can inhibit the pathogen from infecting or transforming cells. (7) Coherence, analogy, biological plausibility. (8) Transmission including identification of the source and means of transmission of the pathogen. (9) Oncogenic mechanisms. (10) Multiple viral and causal factors. (11) Specificity- this criteria was in Hill’s original list but is rarely helpful as many viruses and other pathogens can lead to cancer in different organs.

Hill [8] strongly cautioned against dogmatism.” None of my nine viewpoints can bring indisputable evidence for or against the cause-and-effect hypothesis and none can be required as a *sine qua non* (meaning an essential requirement).

In this current review these criteria could only be fully used with respect to human papilloma viruses because of the limited evidence available for the other pathogens listed above.

### Human papilloma viruses (HPV)

We have recently reviewed the evidence and concluded that it is highly likely that high risk for cancer HPVs have a causal role in prostate cancer [1]. The most important evidence is the demonstration that the prevalence of high-risk HPVs is consistently higher in prostate cancer than in benign prostate controls. This is shown in Table 1 [11–36]. In brief the evidence is as follows:

1. High risk for cancer HPVs have been identified in many countries by a range of methods in normal, benign and malignant prostate tissues [37].
2. In 10 of 27 case control studies conducted with PCR techniques, the prevalence of high-risk HPV DNA was significantly higher in prostate cancers as compared to normal and benign prostate controls (studies in which HPVs were not identified have not been included in Table 1). In these 27 studies there were 399 HPV positive of 1678 prostate cancers (24%) and129 HPV positive of 1331 benign prostate controls (10%) (*p* = 0.001).
3. High risk HPV types 16 and 18 have the capacity to immortalise and transform normal prostate cells into malignant cells [38, 39].
4. HPVs are mainly transmitted by sexual activity [40]. HPVs can be transmitted throughout the body via circulating extra-cellular vesicles and blood [41].
5. High risk HPVs are associated with inflammatory prostatitis which can lead to benign prostate hyperplasia and later prostate cancer [42, 43].
6. High risk HPVs of the same type have been identified in benign prostate tissues 1–11 years before the development of HPV positive prostate cancer in the same patients [44].

**Table 1:** Identification of high risk human papilloma viruses in prostate cancer

Study	Methods	Prostate cancer	Prostate non cancer control	*P* value
McNichol 1991 [11] Canada	PCR	14/27 52%	1/5 20%	0.391 ns
Anwar 1992 [12] Japan	PCR	28/68 41%	0/10 0%	0.002 s
Ibrahim 1992 [13] US	PCR	6/48 13%	2/16 13%	1.00 ns
Rotola 1992 [14] Italy	PCR	6/8 75%	14/17 82%	0.885 ns
Dodd 1993 [15] Canada	PCR	3/7 43%	5/10 50%	0.861 ns
Moyret-Lalle 1995 [16] France	PCR hybridisation	9/17 53%	7/22 32%	0.393 ns
Wideroff 1996 [17] US	PCR	7/56 13%	4/42 10%	0.679 ns
Terris 1997 [18] US	PCR	10/53 19%	5/37 14%	0.569 ns
Serth 1999 [19] Germany	PCR	10/47 21%	1/37 3%	0.026 s
Carozzi 2004 [20] Italy	PCR	14/26 54%	5/25 20%	0.117 ns
Leiros 2005 [21] Argentina	PCR	17/41 42%	0/30 0%	0.002 s
Silvestre 2009 [22] Brazil	HPV genotyping	2/65 3%	0/6 0%	
Martinez-Fierro 2010 [23] Mexico	PCR	11/55 20%	4/75 5%	0.022 s
Aghakhani 2011 [24] Iran	PCR	13/104 13%	8/104 8%	0.298 ns
Chen 2011 [25] Australia	PCR	7/51 14%	3/11 27%	0.715 ns
Tachezy 2012 [26] Czech	PCR	2/95 2%	1/51 2%	
Whitaker 2013 [37] Australia	In situ, standard PCR	29/50 58%	8/50 16%	0.003 s
Ghasemian 2013 [27] Iran	PCR	5/29 17%	8/167 5%	0.025 s
Mokhtari 2013 [28] Iran	IHC	3/30 10%	1/90 1%	
Michopoulou 2014 [29] Greece	PCR	8/50 16%	1/30 3%	0.115 ns
Singh 2015 [30]India	PCR	39/95 41%	11/55 20%	0.056 s
Huang 2016 [31]China	PCR	30/75 40%	9/73 12%	0.003 s
Davila Rodriquez 2016 [32] Mexico	HPV genotyping	12/62 19%	1/25 4%	0.107 ns
Atashafrooz 2016 [33] Iran	PCR	32/200 16%	2/100 2%	0.001 s
Medel Flores 2018 [34] Mexico	PCR	37/189 20%	16/167 10%	0.022 s
Nahand 2020 [35] Iran	PCR	19/58 33%	5/32 16%	0.171 ns
Fatemipour 2021 [36] Iran	PCR	26/72 36%	7/44 16%	0.074 ns

Case control studies with benign prostate tissues as controls. Significant difference between HPV identification in prostate cancer and benign prostate controls *p* =  < 0.05

s, significant; ns, not significant; IHC, immunohistochemistry; PCR, polymerase chain reaction

While the highest prevalence of HPV genital infections occurs in younger people there is an increased prevalence in older age groups (over 55 years) [45, 46]. This increase in older people is unlikely to be due to increased sexual activity. Prostate cancer is much more prevalent in older men. Accordingly there may be an association between older age HPV reactivation and prostate cancer.

The reason for the reactivation of HPVs is not known. An explanation may be the concept of “trained immunity” [47]. This concept involves the long-term reprogramming of innate immune cells, which can be reactivated by stimuli such as infections or chemicals. While this response can be protective against a harmful stimulus, over- reactions such as inflammation can develop. In turn, chronic inflammation can be oncogenic. While there is no direct evidence available with respect to prostate cancer, HPVs can remain dormant in the host cell genome, thus evading the host immune response until they are reactivated [48].

The oncogenic mechanisms for HPV oncogenesis in prostate cancer are not clear and may differ from HPV oncogenesis in cervical cancer. There is evidence that HPV E7 oncogenic proteins may be directly involved early in prostate oncogenesis [17]. HPV infections may have an indirect role by inhibiting the protective function of APOBEC3B enzymes against other virus infections [49, 50].

Effective and safe vaccines are available for the prevention of a wide range of different types of HPV infections [51].

With respect to Silvestre et al. [22], Tachezy et al. [26] and Mokhtari et al. [28] the numbers of positive cases are too few to justify statistical analysis.

### Cutibacterium (Propionbacterium) acnes

*Cutibacterium acnes* (*C. acnes*) are part of the commensal flora of the skin where they colonize hair follicles and sebaceous glands [52]. Different types of *C. acnes* can also cause serious post-operative infections. *Cutibacterium acnes* may also be present in the urogenital tract including the prostate. *Cutibacterium acnes* can damage blood cells, cause host tissue degradation and disrupt cell surface components.

*Cutibacterium acnes* has been identified in prostate cancer tissues. In 2 of 6 case control studies *C. acnes* was significantly more prevalent in prostate cancer than in control benign prostate tissues (Table 2) [53–58]. Most *C. acnes* from prostate cancer tissues differ genetically from common skin *C. acnes* [59]. Alexeyev et al. [53] have identified *C. acnes* in benign prostate tissues taken up to 6 years apart from individual subjects. This indicates that *C. acnes* infection can be chronic and a cause of chronic inflammation. *Cutibacterium acnes* infections induce upregulation of inflammatory genes and cytokine secretion in prostate epithelial cells [60].

**Table 2:** Cutabacterium acnes infections and prostate cancer

Study	Country	Method	Prostate cancer	Prostate non cancer controls	*P* value
Alexeyev 2006 [53]	Sweden	PCR	13/159 8%	6/159 4%	0.119 ns
Sfanos 2007 [54]	US	Bacterial cultures PCR	5/8 63% 10/200 5%	3/8 38%	0.562 ns
Severi 2010 [55]	Australia	Serology	407/808 50%	332/584 57%	0.001 s
Bae 2014 [56]	Japan	IHC	27/28 96%	14/18 78%	0.630 ns
Davidsson 2016 [57]	Sweden	Bacterial cultures	60/100 60%	13/50 26%	0.001 s
Kakegawa 2017 [58]	Japan	IHC	7/44 15%	2/36 5%	0.218 ns

PCR, polymerase chain reaction; IHC, immunohistochemistry; s, significant, ns, not significant

Accordingly *C. acnes* is a candidate pathogen in prostatitis and prostate cancer.

The evidence that antibiotics can control *C. acnes* infections is based on skin infections [61]. Resistance to antibiotics is an increasing problem.

### Escherichia coli

*Escherichia coli* have been consistently identified by PCR and Next Generation Sequencing in prostate cancer and benign prostate tissues [54, 62]. Unfortunately, good controls have not been used in these studies and no case control studies have been identified. A problem in studying *E. coli* and prostate cancer is that biopsies are usually conducted by gaining access to the prostate via the rectum. This can cause contamination of the prostate tissues by rectal located *E. coli*.

*Escherichia coli* is usually a harmless commensal bacteria that colonizes the human gut. However, many different types and strains exist, some of them have virulence properties that can result in inflammation and damage of the prostate. Jain et al. [63] have isolated *E. coli* from benign prostate tissues and demonstrated that this pathogen activated NF-kB and induced damage to normal cultured prostate epithelial cells. NF-kB proteins are activated by carcinogens and are known to be involved in oncogenesis [64]. Hemolysin and necrotizing factor type 1 occur significantly more frequently among *C. coli* isolates causing prostatitis than among those causing cystitis or pyelonephritis [65].

It is considered likely that some types of *E. coli* have causal roles in colon cancer [66]. Accordingly it is possible that *E. coli* can also cause prostate cancer.

### Neisseria gonorrhoea (N. gonorrhoea)

*Neisseria gonorrhoeae* is the well known cause of the sexually transmitted disease gonorrhea [67]. The organism can manipulate the immune response which leads to a lack of protective immunity. Therefore individuals can become repeatedly infected. Gonorrhoea is generally a mucosal infection of the urethra with a pustular discharge. More severe sequalae include salpingitis and pelvic inflammatory disease which may lead to sterility and/or ectopic pregnancy. *Neisseria gonorrhoeae* can cause chronic inflammation of the prostate which in turn can be oncogenic [68]. Gonorrhoea is susceptible to an array of antibiotics. Antibiotic resistance is becoming a major problem.

There have been 22 case control studies in which the prevalence of *N. gonorrhoea* in prostate cancer has been compared to controls (Table 3) [69–90]. In six of these studies it was shown that *N. gonorrhoea* was significantly more prevalent in the prostate cancer cases. In 16 of these studies there was no significant difference been the cases and controls.

**Table 3:** Neisseria gonorrhoea infections and prostate cancer

Study	Country	Method for gonorrhoea	Prostate cancer	Prostate non cancer controls	*P* value
Heshmat 1975 [69]	US	Self report	35/75 47%	29/75 39%	0.486 ns
Baker 1981 [70]	US	Self report	20/44 45%	14/90 16%	0.005 s
Lees 1985 [71]	Canada	Clinical records	13/83 16%	30/166 18%	0.689 ns
Mishina 1985 [72]	Japan	Self report	26/100 26%	21/100 21%	0.512 ns
Checkoway 1987 [73]	US	Self report	6/40 15%	8/64 13%	0.752 ns
Honda 1988 [74]	US	Self report	33/216 15%	25/216 12%	0.324 ns
Oishi 1989 [75]	Japan	Self report	9/100 9%	35/200 18%	0.086 ns
La Vecchia 1993 [76]	Italy	Self report	3/271 1%	14/685 2%	0.837 ns
Hiatt 1994 [77]	US	Medical record	9/238 4%	6/238 3%	0.446 ns
Ilic 1996 [78]	Serbia	Self report	4/101 4%	0/202 0%	0.016 s
Hsieh 1999 [79]	Greece	Self report	39/320 12%	22/246 9%	0.267 ns
Hayes 2000 [80]	US Blacks US Whites	Self report Self report	115/477 24% 15/501 3%	103/588 18% 18/711 3%	0.032 s 0.623 ns
Rosenblatt 2001 [81]	US	Self report	85/753 11%	67/703 10%	0.623 ns
Sanderson 2004 [82]	US	Self report	43/401 11%	33/389 8%	0.332 ns
Patel 2005 [83]	US blacks US whites	Self report	139/353 40% 16/347 5%	94/257 37% 18/347 5%	0.449 ns 0.738 ns
Pelucci 2006 [84]	Italy	Self report	4/280 1%	17/689 3%	0.611 ns
Sarma 2006 [85]	US blacks	Self report	84/129 66%	369/703 53%	0.001 s
Sutcliffe 2006 [86]	US	Self report	55/8770 1%	1999/291,519 1%	0.853 ns
Huang 2008 [87]	US blacks US whites	Self report	30/98 31% 30/762 4%	115/353 33% 26/907 3%	0.753 ns 0.832 ns
Hrbacek 2011 [88]	Czech Republic	Serology	20/328 6%	6/105 6%	0.917 ns
Vazquez-Salas 2015 [89]	Mexico	Self report	81/402 20%	46/805 6%	0.001 s
Wang 2017 [90]	Taiwan	Laboratory	6/355 2%	5/1420 0.4%	0.001 s

s, significant; ns, non-significant

There is a possible explanation for these conflicting data, namely that sexually transmitted diseases are frequently due to multiple pathogens. In the meta-analysis by Taylor et al. [91] there were significant correlations between both *N. gonorrhoea* and HPVs and increased prevalence of prostate cancer (odds ratios gonorrhoea 1.35, HPV 1.39). It is possible that high risk HPVs were the cause of prostate cancer in these studies and that *N. gonorrhoea* was also present but not oncogenic.

### Herpes viruses

#### Herpes simplex

Herpes simplex virus 1 (HSV-1) commonly causes infections of the mouth (cold sores).

HSV-2 is associated with anogenital infections and is a sexually transmitted infection.

Both virus types can cause both kinds of infection. Infections due to herpes simplex do not usually confer immunity. No vaccines are currently available.

In four of 12 studies Herpes simplex 1 or 2 were significantly more prevalent in the prostate cancer cases (Table 4) [70, 87, 88, 92–98]. Dennis et al. demonstrated that herpes simplex 2 could be identified in prostate cancer tissues over a period of 8 years [98]. These findings suggest that if herpes simplex has an oncogenic capacity there may be a long latency period for prostate cancer development after HSV-2 infection.

**Table 4:** Herpes simplex virus infections and prostate cancer

Study	Country	Method	Prostate cancer	Prostate non cancer controls	*P* value
Baker 1981 HSV 2 [70]	US	Immunofluorescent Tissues	34/50 68%	81/159 51%	0.001 s
Luleci 1981 HSV 2 [92]	Turkey	Serology	14/16 88%	22/35 63%	0.064 ns
Boldogh 1983 [93]	US	ISH	2/10 20%	1/22 5%	0.012 s
Haid 1984 HSV 2 [94]	US	Immunofluorescent tissues	7/27 26%	8/33 24%	0.668 ns
Leskinen 2003 HSV 1,2 [95]	Finland	PCR tissues	0/10	0/10	
Korodi 2005 HSV 2 [96]	Finland	Serology	11/163 7%	20/288 7%	0.721 ns
Bergh 2007 HSV 1,2 [97]	Sweden	PCR tissues	0/201	0/201	
Huang 2008 HSV 2 [87]	US whites US blacks	Serology Serology	70/765 9% 55/103 53%	89/915 10% 180/367 49%	0.729 ns 0.342 ns
Dennis 2009 HSV 2 [98]	US	Serology latent period tests 1 year 8 year	26/55 47% 20/56 36%	47/139 34% 35/156 22%	0.002 s 0.050 s
Hrbacek 2011 HSV 2 [88]	Czech	Serology	313/329 95%	99/105 94%	0.955 ns

s, significant; ns, non significant

Acyclovir has been successfully used to treat genital herpes simplex infections [99].

### Epstein Barr virus (EBV) (Herpes virus 4)

Cancers including breast and prostate cancer [1, 100].

There have been four case control studies of EBV and prostate cancer. In one study by Sfanos et al. [54], EBV was significantly more prevalent in prostate cancer compared to controls (Table 5) [54, 97, 100, 101].

**Table 5:** Epstein Barr virus (herpes virus 4) infections and prostate cancer

Study	Country	Method	Prostate cancer	Prostate non cancer controls	*P* value
Grinstein 2002 [101]	Argentina	IHC	7/19 37%	0/10 0%	0.089 ns
Bergh 2007 [97]	Sweden	PCR tissues	15/115 9%	14/115 9%	0.861 ns
Sfanos 2008 [54]	US	PCR tissues	16/200 8%	5/200 10%	0.019 s
Nahand 2021 [100]	Iran	PCR tissues	10/67 15%	3/40 8%	0.310 ns

s, significant; ns, non significant

The effectiveness of antiviral agents (acyclovir, valomaciclovir and valacyclovir) in acute infectious mononucleosis is uncertain [99, 102].

### Cytomegalovirus (CMV) (herpes virus 5)

Human CMV is present in over 80% of most populations. Transmission can occur during foetal life, via breast milk, saliva and during sexual activities. Human CMV infections in healthy people are mostly mild or without symptoms. In contrast, CMV can cause serious defects during foetal life and life threatening illness among immunocompromised patients such as transplant recipients and patients with AIDS [103].

As shown in Table 6 [23, 87, 93, 104, 105] in four of five case control studies there were no significant differences between the prevalence of CMV in prostate cancers and controls. In one study CMV was identified in the controls but not in prostate cancers [23].

**Table 6:** Cytomegalovirus infections and prostate cancer

Study	Country	Method	Prostate cancer	Prostate non cancer controls	*P* value
Boldogh 1983 [93]	US	ISH	4/10 40%	5/22 23%	0.461 ns
Eizuru 1983 [104]	US	ISH	0/5 0%	0/12 0%	0.554 ns
Samanta 2003 [105]	US	IHC	17/17 100%	5/5 100%	1.000 ns
Huang 2008 [87]	US whites US blacks	Serology	538/769 70% 90/103 87%	626/920 68% 328/369 89%	0.452 ns 0.439 ns
Martinez- Fierro 2010 [23]	Mexico	PCR	0/55 0%	6/75 8%	0.037 s Control > cancer

ISH, in-situ hybridisation; IHC, immunohistochemistry; s, significant; ns, non significant

### Chlamydia trachomatis (C. trachomatis)

*Chlamydia trachomatis* is a common, sexually transmitted bacteria. *Chlamydia trachomatis* initiates and can maintain inflammation and persistent infection including prostatitis [105]. Human prostate cancer epithelial cells are susceptible to *C. trachomatis* infection and initiate inflammation [106, 107]. As inflammation is associated with prostate cancer it has been hypothesized that *C. trachomatis* could have a causal role.

However, as shown in Table 7 [81, 87, 88, 98, 106, 108–110] in eight case control studies there were no positive associations between *C. trachomatis* infections and prostate cancer. On the other hand, all these studies are based on serology, and it is possible that these case control studies are misleading as *C. trachomatis* may be causing chronic infection in the prostate leading to prostate cancer. This would lead to positive antibodies in both benign prostate controls and prostate cancer.

**Table 7:** Chlamydia trachomatis infections and prostate cancer

Study	Country	Method	Prostate cancer	Prostate non cancer controls	*P* value
Dillner 1998 [106]	Finland	Serology	18/165 11%	31/290 11%	0.948 ns
Rosenblatt 2001 [81]	US	Self report	5/748 1%	9/694 1%	0.229 ns
Antilla 2005 [108]	Finland, Sweden, Norway	Serology	55/738 8%	238/2271 11%	0.030 s Control > cancer
Sutcliffe 2007 [109]	US	Serology	28/655 4%	24/655 4%	0.990 ns
Huang 2008 [87]	US black US white	Serology	37/103 36% 86/765 11%	131/367 36% 89/915 10%	0.977 ns 0.362 ns
Dennis 2009 [98]	US	Serology	39/267 15%	31/267 12%	0.369 ns
Hrbacek 2011 [88]	Czech Republic	Serology	18/329 6%	12/105 11%	0.054 s Control > cancer
Lumme 2016 [110]	Finland	Serology	51/6699 7%	224/2132 11%	0.002 s Control > cancer

s, significant; ns, non significant; − s, statistically negative significant

Azithromycin and Doxycycline antibiotics appear to be effective in the treatment of sexually transmitted *C. trachomatis* [111].

### Trichomonas vaginalis (T.vaginalis)

*Trichomonas vaginalis* is a common protozoan infection frequently transmitted during sexual activities [112]. *Trichomonas vaginalis* in men is usually asymptomatic but may cause urethritis, prostatitis, epididymitis and infertility [113].

As shown in Table 8 [86, 114–121] in eight of nine case control studies there is no increase in risk of prostate cancer in association with *T. vaginalis* infections. In two studies positive antibodies were higher in the controls than the cancer. These nine studies were all based on serology and involved a high number of subjects.

**Table 8:** Trichomonas vaginalis infections and prostate cancer

Study	Country	Method	Prostate cancer	Prostate non cancer controls	*P* value
Sutcliffe 2006 [86]	US	Serology	87/691 13%	65/691 9%	0.090 ns
Sutcliffe 2009 [114]	US	Serology	132/616 22%	153/616 25%	0.262 ns Control > cancer
Stark 2009 [115]	US	Serology	165/673 25%	144/673 21%	0.402 ns
Chen 2013 [116]	US	Serology	87/603 14%	65/627 10%	0.056 ns
Shui 2016 [117]	US	Serology	24/122 20%	42/139 30%	0.130 ns Control > cancer
Fowke 2016 [118]	US	Serology	69/296 23%	124/585 21%	0.567 ns
Marous 2017 [119]	US whites US blacks	Serology	84/777 11% 43/158 27%	33/405 8% 75/280 27%	0.127 ns 0.943 ns
Kim 2019 [120]	Korea	Serology	9/44 20%	1/58 2%	0.001 s
Saleh 2021 [121]	Egypt	Serology	24/126 19%	10/120 8%	0.015 s

s, significant; ns, non significant

In a large serology based study by Tsang et al. [122] there was no increase in prostate cancer deaths associated with *T. vaginalis*. This finding makes it unlikely that *T. vaginalis* is associated with prostate cancer.

The 5-nitroimidazoles (metronidazole, tinidazole, secnidazole) are the only class of antimicrobials effective against *T. vaginalis* [113]. Unfortunately, there is growing concern over drug resistance with metronidazole.

### Mycoplasma

Mycoplasma bacteria frequently infect prostate tissues and prostate cancer. The most common are *M. hominus*, *M. ureaplasma* and *M. hyorhinus* [123]. A recent meta-analysis showed that Mycoplasma bacterial infections were 2.24 times more frequent in patients with prostate cancer as compared to benign prostate hyperplasia [124]. These data are shown in Table 9 [88, 123, 125–129].

**Table 9:** Mycoplasma infections and prostate cancer

Study	Country	Methods	Prostate cancer	Prostate non cancer controls	*P* value
Hrbacek 2011 M. hominis [88]	Czech Republic	Serology	60/330 18%	16/107 14%	0.518 ns
Hrbacek 2011 M. urealyticum [88]	Czech Republic	Serology	64/328 20%	11/105 11%	0.068 ns
Barykova 2011 M. hominis [123]	Russia US	Serology PCR	28/125 22% 5/27 19%	0/27 0% 4/31 13%	0.023 s
Urbanek 2011 M. hyorhinus [125]	US	Serology	59/114 52%	38/105 36%	0.279 ns
Erturhan 2013 [126]	Turkey	PCR	11/31 35%	0/31 0%	0.004 s
Yow 2014 M. genitalium [127]	Australia	PCR	9/115 8%	1/51 2%	0.163 ns
Miyake 2019 M. genitalium [128]	Japan	PCR	18/45 40%	6/33 18%	0.127 ns
Saadat 2020 M. Hominis [129]	Iran	PCR	8/61 13%	0/70 0%	0.003 s

s, significant; ns, non significant

Of particular interest are the studies based on PCR analyses of tissues as compared to studies based on serology. Three of the PCR studies with positive results were significant, and two showed a trend that Mycoplasma infections were more frequent in prostate cancers than benign prostate controls. Accordingly, it is possible that Mycoplasma bacteria may have a role in prostate cancer. However additional evidence is required.

Antibiotics can be effective in treating Mycoplasma bacterial infections. Unfortunately, resistance to antibiotic treatment is emerging [130].

### Polyoma viruses (hPy)

The two human polyomaviruses (hPy), BK virus (BKV), and JC virus (JCV), are commonly present in human populations. Infections usually occurs in childhood but rarely cause clinical symptoms. In immunocompromised patients JCV can cause serious neurodegenerative conditions. There is no direct evidence that hPy viruses are oncogenic [131].

We have identified 11 case control studies of BKV and JCV and their associations with prostate cancer in which polyoma viruses were identified (Table 10) [97, 132–139]. In two small studies based on PCR there was a significant association with prostate cancer. There were no significant associations in 9 studies.

**Table 10:** Polyoma BKV, JCV prostate cancer

Study	Country	Method	Prostate cancer	Prostate non cancer controls	*P* value
Monini 1995 BKV [132]	Italy	PCR	4/7 57%	11/19 58%	0.986 ns Benign > cancer
Zambrano 2002 BKV [133]	US	PCR	2/8 25%	1/11 9%	0.427 ns
Zambrano 2002 JCV [133]	US	PCR	3/8 38%	4/11 36%	0.973 ns
Bergh 2007 JCV [97]	Sweden	PCR	3/159 2%	6/159 4%	0.324 ns Benign > cancer
Lau 2007 BKV [134]	US	ISH	2/30 7%	4/30 13%	0.481 ns Benign > cancer
Das 2008 BKV [135]	US	ISH	11/14 79%	4/15 27%	0.090 ns
Russo 2008 BKV [136]	Italy	IHC	20/26 77%	0/12 0%	0.004 s
Delbue 2013 BKV [137]	Italy	PCR	18/56 32%	15/68 22%	0.318 ns
Delbue 2013 JCV [137]	Italy	PCR	16/56 28%	16/68 24%	0.624 ns
Taghavi 2015 BKV [138]	Iran	PCR	17/60 28%	9/60 15%	0.154 ns
Gorish 2019 BKV [139]	Sudan	Immunofluoresence PCR	17/55 30% 16/17 94%	4/55 7% 2/4 50%	0.009 s 0.493 ns

IHC, immunohistochemistry; ISH, in situ hybridisation; s, significant; ns, non significant

Accordingly it is unlikely that these polyomaviruses have causal roles in prostate cancer.

### Fungal prostatitis

Infections of the prostate by several fungi are the unusual cause of prostatitis. These fungi include Blastomycosis, Candida albicans and Cryptococcus [140]. There is no evidence that these fungi are associated with prostate cancer. However, there must be suspicions about any pathogen which leads to chronic inflammation.

### Mouse mammary tumour virus (MMTV)

MMTV is the proven cause of breast cancer in mice. There is compelling evidence that MMTV—like viruses are also causal in human breast cancer [7]. MMTV has been identified in prostate glands of mice [141]. MMTV—like viruses have been identified in human prostate cancers [6]. However, no studies have been conducted to determine if MMTV is causal in human prostate cancer.

### Human immunodeficiency virus (HIV)

Compared to the general population, people living with HIV have a lower prevalence of prostate cancer [142, 143]. This is probably due to the suppression of immune related B and T cells associated with both HIV and MMTV infections.

### The gut microbiome and prostate cancer

The gut microbiome may also play an indirect role in various cancers [144]. In a study which compared the gut microbiota in men with prostate cancer and benign controls there was a significant difference in gut microbiol composition [145]. The meaning of these observations is not known.

## Discussion

High risk human papilloma viruses are the only pathogens for which there is sufficient evidence to indicate a probable causal role in prostate cancer. Fortunately, there are safe and effective vaccines available to prevent HPV infections [146].

Other pathogens may have roles in prostate cancer but the evidence is limited. These include Cutibacterium acnes, Neisseria gonorrhoea, Herpes simplex, Epstein Barr virus, and Mycoplasmas. In our view it is unlikely that Cytomegalovirus, Trichomonis vaginalis, Chlamydia trachomonis, Polyoma viruses, Human immunodeficiency virus and fungi have causal roles in prostate cancer.

HPVs are the only pathogen considered in this review which have a proven oncogenic capacity. However, in its acute stage it is unlikely that an HPV infection leads to prostate cancer as HPV infections are common in young men and prostate cancer occurs mainly in older men. On the other hand, as considered above, the influence of HPV may be reactivated and lead to prostate oncogenesis via long-term reprogramming of innate immune cells.

While the oncogenic mechanisms probably differ between these pathogens, of particular relevance is the potential role of inflammation in prostate cancer. Different pathogens may each cause chronic inflammation. Multiple pathogens are frequently present in prostate tissues and chronic exposure can lead to chronic inflammation and ultimately to prostate cancer. The relevant evidence has been reviewed in detail by De Bono et al. [147] and Gobel et al. [148].

A precise mechanism linking inflammation to cancer is the nuclear transcription factor “kappa-light-chain-enhancer” of B-cells known as NF-kB. This is a protein activated by many carcinogens. It controls genes commonly associated with oncogenesis [64]. Almost all infectious agents linked with cancer activate NF-nB. This has been confirmed experimentally in mice by the inactivation of NF-kB which reduced inflammation initiated cancer formation [149]. Infectious pathogens can activate inflammatory pathways which lead to genomic instability in tissue cells which in turn lead to malignant transformation. HPV, human herpes virus, and EBV, have been specifically shown to activate NF-kB. Confirmation of this evidence has been provided by the reduction in risk of cancer by anti-inflammatory agents such as aspirin [150].

## Conclusions and actions

The most influential cause of prostate cancer appears to be infection induced chronic inflammation.

Given the high prevalence of prostate cancer it is important for action to can be taken without waiting for additional conclusive evidence. These include:

1. Encouragement of all boys (as well as girls) to have HPV vaccines
2. The vigorous use of antibiotics to treat all bacterial pathogens identified in the urogenital tract
3. The use of antiviral medications to control herpes infections
4. Education about safe sexual practices

## Data Availability

Not applicable.

## Abbreviations

HPV: Human papilloma viruses
*C. acnes*: *Cutibacterium acnes*
*N. gonorrhoea*: *Neisseria gonorrhoea*
HSV: Herpes simplex virus
EBV: Epstein Barr virus
CMV: Cytomegalovirus
*C. trachomatis*: *Chlamydia trachomatis*
*T. vaginalis*: *Trichomonas vaginalis*
hPy: Polyoma virus
MMTV: Mouse mammary tumour virus
HIV: Human immunodeficiency virus
